# Transcriptional and Proteomic Characterization of Telomere-Induced Senescence in a Human Alveolar Epithelial Cell Line

**DOI:** 10.3389/fmed.2021.600626

**Published:** 2021-02-09

**Authors:** Daniel I. Sullivan, Mao Jiang, Angela M. Hinchie, Mark G. Roth, Harinath Bahudhanapati, Mehdi Nouraie, Jie Liu, John F. McDyer, Rama K. Mallampalli, Yingze Zhang, Daniel J. Kass, Toren Finkel, Jonathan K. Alder

**Affiliations:** ^1^Department of Medicine, Division of Pulmonary, Allergy and Critical Care Medicine, University of Pittsburgh, Pittsburgh, PA, United States; ^2^Dorothy P. and Richard P. Simmons Center for Interstitial Lung Disease, Pittsburgh, PA, United States; ^3^The Third Xiangya Hospital, Central South University, Changsha, China; ^4^Aging Institute, University of Pittsburgh, Pittsburgh, PA, United States; ^5^University of Pittsburgh Medical Center, Pittsburgh, PA, United States; ^6^Division of Cardiology, Department of Medicine, University of Pittsburgh, Pittsburgh, PA, United States; ^7^Department of Internal Medicine, The Ohio State University, Columbus, OH, United States

**Keywords:** telomerase, SASP, secretome, IPF, mass spectrometry, biomarker, A549, aging

## Abstract

Cellular senescence due to telomere dysfunction has been hypothesized to play a role in age-associated diseases including idiopathic pulmonary fibrosis (IPF). It has been postulated that paracrine mediators originating from senescent alveolar epithelia signal to surrounding mesenchymal cells and contribute to disease pathogenesis. However, murine models of telomere-induced alveolar epithelial senescence fail to display the canonical senescence-associated secretory phenotype (SASP) that is observed in senescent human cells. In an effort to understand human-specific responses to telomere dysfunction, we modeled telomere dysfunction-induced senescence in a human alveolar epithelial cell line. We hypothesized that this system would enable us to probe for differences in transcriptional and proteomic senescence pathways *in vitro* and to identify novel secreted protein (secretome) changes that potentially contribute to the pathogenesis of IPF. Following induction of telomere dysfunction, a robust senescence phenotype was observed. RNA-seq analysis of the senescent cells revealed the SASP and comparisons to previous murine data highlighted differences in response to telomere dysfunction. We conducted a proteomic analysis of the senescent cells using a novel biotin ligase capable of labeling secreted proteins. Candidate biomarkers selected from our transcriptional and secretome data were then evaluated in IPF and control patient plasma. Four novel proteins were found to be differentially expressed between the patient groups: stanniocalcin-1, contactin-1, tenascin C, and total inhibin. Our data show that human telomere-induced, alveolar epithelial senescence results in a transcriptional SASP that is distinct from that seen in analogous murine cells. Our findings suggest that studies in animal models should be carefully validated given the possibility of species-specific responses to telomere dysfunction. We also describe a pragmatic approach for the study of the consequences of telomere-induced alveolar epithelial cell senescence in humans.

## Introduction

Idiopathic pulmonary fibrosis (IPF) is a progressive, fibrosing lung disease whose incidence increases with age (1, 2). The average age at presentation is 66 years, and two thirds of all diagnoses are made after age 60 (3). It is an uncommon disease that will almost certainly become more common as our population ages (4). Currently, the prognosis for IPF is often worse than many cancers (5, 6), and the two drugs that exist to treat this disease have only modest effects on disease progression (7, 8). A more complete understanding of the pathogenesis of this disease is essential to the development of novel therapeutics for IPF. Recently, a greater emphasis has been placed on the contribution of the alveolar epithelial cell to the development of this disease (9–14).

Alveolar epithelial cells, while incapable of forming scar tissue themselves, are held to play a causal role in IPF pathogenesis (15–19). Epithelial cell senescence as a result of telomere dysfunction is one component of the alveolar epithelial cell theory of IPF (9). Short telomeres have been identified as a risk factor for IPF (20–28), and IPF is the most common clinical manifestation of patients with mutations in telomere maintenance genes (29). Approximately half of sporadic and >60% of familial cases of IPF have short telomeres (20). When telomeres reach a critically short length, affected cells will either apoptose or become senescent (30). We previously developed a murine model of telomere-dysfunction and found that type 2 epithelial cells (AEC2s)—the principal alveolar progenitor cells—preferentially become senescent in the setting of telomere dysfunction (9). The AEC2s lost their regenerative capacity and rendered the host exquisitely sensitive to pulmonary injury. Additionally, secondary mesenchymal effects were seen *in vivo* that were hypothesized to be due to AEC2s adopting the senescence-associated secretory phenotype (SASP) (9)—a set of characteristic, paracrine signaling pathways (31) that is believed to play a role in the progression of fibrotic lung disease (32). However, while these cells displayed many of the characteristic findings of senescence, few SASP genes were upregulated in this study (9).

The absence of this phenotype led us to hypothesize that the response to cellular senescence may be species-specific, and we sought to develop a human lung epithelial cell model to examine the consequences of telomere dysfunction in a more clinically relevant cell type. We selected the p53 competent, alveolar epithelial-like cell line, A549 (33). As in our prior mouse model, we chose telomeric repeat-binding factor 2 (TERF2) as our target of intervention. As a component of the shelterin complex, TERF2 serves to prevent telomeric ends from being recognized as double-stranded DNA breaks (34). Given the known poor correlation between mRNA and protein levels in human tissues (35), we also employed a novel endoplasmic reticulum (ER) targeted biotin ligase to characterize the secreted protein (secretome) changes induced by cellular senescence. We hypothesized that this combined approach would enable the most complete characterization of human telomere-induced alveolar epithelial senescence and provide the greatest opportunity to identify relevant secreted proteins.

Herein we show that telomere dysfunction in a human alveolar epithelial-like cell line leads to cellular senescence and an upregulation in transcriptional and proteomic SASP. Our results suggest that the consequences of telomere dysfunction may be species-specific and perhaps cell-type specific. We also introduce a set of adaptable tools for the induction of senescence and study of its effects on protein secretion in a variety of cell types.

## Methods

### Tissue Culture and Generation of Stable Cell Lines

A549 cells were acquired from ATCC and cultured in DMEM supplemented with 10% fetal bovine serum and penicillin (120 U/mL), streptomycin (100 mcg/mL), and L-glutamine (2 mM). A construct for conditional induction of telomere dysfunction was generated by cloning a truncated version of human TRF2 protein that lacks the N-terminal basic domain and C-terminal Myb domains (36) into the lentiviral vector pCW57-GFP-2A-MCS, a gift from Adam Karpf (Addgene plasmid #71783) (37). Lentiviral particles were generated as described previously (38) and used to transduce low-passage A549 cells. Following transduction, individual clones of cells were selected that showed strong expression of the transgene in the presence of 2 μg/mL doxycycline. Proliferation studies were carried out by plating three independent cultures of each cell line and enumerating cells at each passage. The total number of cells were log2 transformed and plotted against time. Fresh doxycycline (2 μg/mL final concentration) was added at each passage. Clonogenic assays were performed by plating 1,000 cells in 10 cm dishes and enumerating colonies following staining with crystal violet after 12 days in culture. Media was replaced with fresh doxycycline (2 μg/mL final concentration) every 48 h during the course of the experiment. Senescence-associated beta-galactosidase (SA-βgal) was stained according to manufacturer's protocol (Cell Signaling Technologies). Proximity ligation experiments were carried out by expressing a modified biotin ligase (BioID2) (39) that had been targeted to the endoplasmic reticulum (ER) by addition of a N-terminal IgK signal sequence and C-terminal ER retention sequence (KDEL) (40).

### Western Blots and Immunoprecipitation

Western blots were performed following standard procedures and employed antibodies specific for Flag epitope (M2, Millipore Sigma), V5 (Thermo Fisher), HA (Millipore Sigma), p21 (Cell Signaling Technologies), and GAPDH (BioRad). Briefly, cells were lysed in RIPA buffer containing protease and phosphatase inhibitors (MiniComplete, Roche). Following protein quantitation, 20–40 μg of protein or 18 μL of media were separated under reducing conditions using SDS-PAGE and transferred to PVDF membranes. Proteins were blotted with antibodies specific for the desired protein and visualized on a ChemiDoc MP gel documentation system (BioRad). Immunoprecipitation of V5-tagged proteins was accomplished by incubating media containing V5-tagged proteins with Anti-V5 agarose (Millipore Sigma) according to the manufacturer's instructions.

### Transcriptional Profiling and Analysis

Total RNA was isolated from biologic replicates (*n* = 3) of cultured cells using RNAeasy kits (Qiagen) according to manufacturer's protocol and sent for library preparation, sequencing, quality control, alignment, differential expression analysis, and preliminary enrichment analysis at Novogene (Sacramento, CA). Approximately 20 million paired-end fragments were sequenced for each sample. The raw data have been deposited in NCBI's Gene Expression Omnibus (41) GSE155941. Expression data from senescent murine AEC2s were obtained from GSE56892 (9). Additional enrichment analyses were conducted using Ingenuity Pathway Analysis (Qiagen), Gene Ontology (GO), and Kyoto Encyclopedia of Genes and Genomes (KEGG). Differential expression of several genes was confirmed using quantitative real-time PCR with primers specific for the selected genes.

### Proximity Labeling and Mass Spectrometry

Validation of the BioID2 targeting and function was accomplished by transfecting cells stably expressing ER-targeted BioID2 with a plasmid encoding V5-tagged human *SFTPA2* cDNA (pCDNA3-V5-SFTPA2) (14). Eighteen hours after transfection, media was supplemented with biotin (100 μM). The next day, cells and media were collected for western blot analysis. V5-tagged SFTPA2 was immunoprecipitated with anti-V5 resin (Millipore). Detection of biotinylated proteins was accomplished by incubating membranes with streptavidin conjugated to horseradish peroxidase (Strep-HRP) and developing the membranes according to the manufacture's protocol (Vector Laboratories). The unbiased proteomic screen of telomere dysfunction-induced senescence-related changes was carried out by comparing TRF2-DN-BioID2 and TRF2-DN-BioID2+Doxycline. Four days after addition of doxycycline, biotin was added to the media. Eight hours later, cells were washed to remove excess biotin and fresh media was added. Twenty-four hours later, the supernatant was collected, and biotinylated proteins were purified by incubating media with streptavidin coated beads according to the manufacturer's protocol (Dynabeads MyOne Streptavidin C1; Invitrogen). Half of the sample was eluted at 95°C for 10 min in loading buffer and run on a 4–15% SDS-PAGE gel to evaluate yield of recovered protein. The remainder of the protein coated beads were sent to MS Bioworks (Ann Arbor, MI) for mass spectrometry analysis where they were eluted, gel separated, split into 10 samples based on molecular weight, and digested samples were analyzed by nano LC/MS/MS with a Waters NanoAcquity HPLC system interfaced with a ThermoFisher Q Exactive mass spectrometer. A single sample was submitted for each condition. Data were searched using Mascot (Matrix Science) and parsed into Scaffold™ (Proteome Software Inc.) for validation, filtering and to create a non-redundant list per sample. Data were filtered using a 1% protein and peptide level false discovery rate (FDR) and by requiring at least two unique peptides per protein.

### Immunostaining and Imaging

Cells were grown on coverslips and fixed in 2% PFA for 10 min. Following fixation cells were washed, permeabilized with Triton X-100, and blocked with goat serum. Coverslips were incubated with primary antibodies including rat anti-HA (Millipore Sigma) and rabbit anti-calnexin (Cell Signaling). Proteins were visualized with secondary antibodies conjugated to Alexa 594 and Alexa 647 (Thermo Fisher). Nuclei were stained with 4′,6-diamidino-2-phenylindole (DAPI). Images were obtained at the Center for Biologic Imaging at the University of Pittsburgh on an Olympus FluoView Confocal microscope. Brightfield photomicrographs were captured on an Observer A.1 (Zeiss) equipped with AxioCam MRc camera.

### Multiplex Screen of Serum Biomarkers

Transcriptional and proteomic data were used to rationally select 17 candidate biomarkers for evaluation in a discovery cohort of control (*n* = 30) and IPF (*n* = 50) patients. Plasma samples from these patients were evaluated using Luminex® panels purchased from R&D systems. Candidates biomarkers were selected based on their differential expression in our current study, the availability of compatible commercial assays to simultaneously measure several proteins, and their dilution compatibility with the chosen assay. For this initial study, we only evaluated proteins that had previously been reported to be detectable in human plasma samples. Panels were analyzed on a Bio-Plex reader (Bio-Rad) according to the manufacturer's protocol. Biomarkers selected from the discovery round were evaluated for correlations with baseline pulmonary function studies in IPF patients.

### Human Subjects

All studies were approved by the University of Pittsburgh Institutional Review Board and the Committee for Oversight of Research and Clinical Training Involving Decedents. All subjects provided written, informed consent before enrollment in the research study. IPF subjects were recruited from the Simmons Center for Interstitial Lung Diseases at the University of Pittsburgh Medical Center. Clinical, physiologic, and high-resolution computed tomography studies of these patients supported the diagnosis of IPF. Patients fulfilled the criteria of the American Thoracic Society and European Respiratory Society for the diagnosis of IPF at the time of diagnosis (3, 42). Patients with known causes of interstitial lung disease were excluded. Control patients consisted of unrelated healthy subjects, randomly recruited from the University of Pittsburgh Medical Center, and had no self-reported advanced lung diseases.

### Statistical Analysis

All cellular images shown are representative of multiple experiments. RNA-seq differential expression analysis was performed using the DESeq2 R package (43). Fisher's exact test was used for differential expression analysis of mass spectrometry identified proteins. Simple linear regression was used for differential protein vs. RNA correlation. Control vs. IPF plasma protein levels were evaluated using Welch's *t*-test of significance. The Benjamini-Hochberg procedure was used for all corrections of multiple testing. Pearson correlation coefficients for IPF patient baseline PFT values were calculated using square-root-transformed protein levels.

## Results

### Induction of Telomere Dysfunction Drives Senescence of Human Lung Epithelial-Like Cells

**Figure 1:**
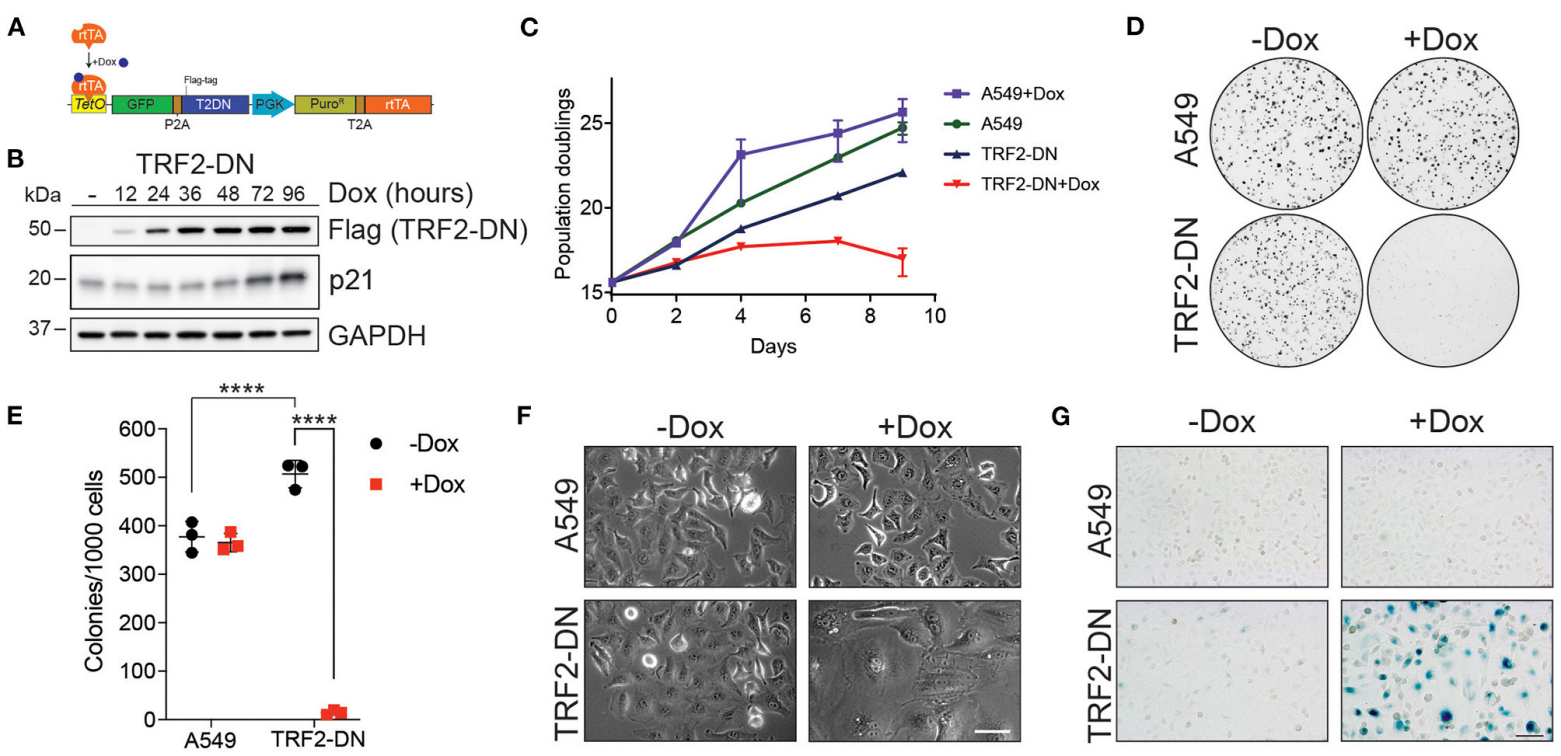
Induction of telomere dysfunction causes senescence of a human lung epithelial cell line. **(A)** Schematic of all-in-one lentivirus expressing green fluorescent protein (GFP), dominant negative TRF2 (T2DN; TRF2-DN hereafter), puromycin N-acetyl transferase (Puro^R^), and reverse Tet repressor (rtTA). GFP and TRF2-DN are expressed by a doxycycline-inducible promoter while Puro^R^ and rtTA are expressed by the constitutive phosphoglycerate kinase promoter (PGK). **(B)** Time course of protein expression following induction with doxycycline (dox). Relative protein levels of TRF2-DN (flag), p21, and GAPDH (load control) are shown. **(C)** Relative proliferation of A549 and A549 cells stably expressing TRF2-DN. Viable cells were counted with trypan blue staining following induction with doxycycline. Mean and standard error of the mean are shown for each count (*n* = 3). **(D)** Representative images of crystal violet stained clonogenic assays. Media was changed every 2–3 days after plating 1,000 cells and plates were imaged after 12 days. **(E)** Quantitation of visible colonies from **(D)**. Mean and standard deviation are shown. **(F)** Photomicrograph of A549 and TRF2-DN cells using phase-contrast microscopy. Images were taken 9 days after induction of TRF2-DN with Dox. **(G)** SA-βgal activity of cells shown in **(F)**. Scale bar in **(F,G)** is 100 microns. ****P < 0.0001, one-way ANOVA and Tukey *post hoc* test.

In order to create a model of human, telomere dysfunction-induced, alveolar epithelial cell senescence, we generated a stable A549 cell line that conditionally expressed a dominant negative form of human TRF2 (TRF2-DN) (Figure 1A). Expression of TRF2-DN disrupts shelterin function (36) and leads to telomere uncapping and a subsequent DNA damage response. Conditional induction of TRF2-DN protein led to upregulation of cyclin dependent kinase inhibitor CDKN1A (p21) and halted proliferation (Figures 1B,C). We noted that cells that expressed the TRF2-DN transgene consistently proliferated at a lower rate compared to untransduced cells, likely due to low-level baseline expression of the transgene. TRF2-DN expression limited the clonogenic potential of A549 cells and triggered morphologic changes consistent with the induction of senescence (Figures 1D,F). The apparent increase in colony number for untreated TRF2-DN in Figure 1E is due to smaller, unmerged colonies (data not shown). Consistent with the above findings, cells expressing the TRF2-DN stained strongly for SA-βgal (Figure 1G). Together, these data suggest that disruption of shelterin function is sufficient to drive cellular senescence in A549 cells.

### Comprehensive Transcriptional Profile of Senescent A549 Cells

**Figure 2:**
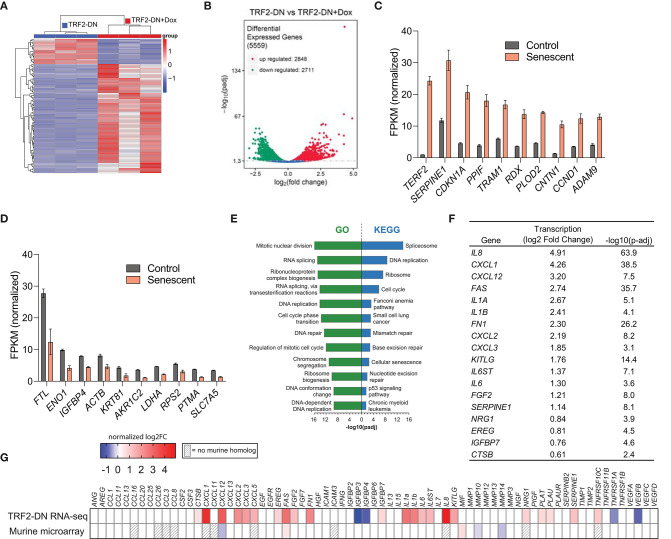
Dysfunctional telomeres drive senescence-associated transcriptional changes in A549 cells. **(A)** Hierarchical cluster analysis of differentially expressed genes identified using RNA-seq of TRF2-DN and TRF2-DN+Dox 9 days after induction (*n* = 3 per group). Red indicates up-regulated transcripts; blue indicates down-regulated transcripts. The fold-change based on color is shown in the key. **(B)** Volcano plot depicting 5,559 differentially expressed genes. Significance defined as –log_10_(p-adj) > 1.3. The 10 most upregulated **(C)** and downregulated **(D)** transcripts between TRF2-DN (control) and TRF2-DN+Dox (senescent) cells. Mean and standard deviation are shown. All differences are statistically significant, but *P*-values are not shown for clarity. **(E)** Gene Ontology (GO) and Kyoto Encyclopedia of Genes and Genomes (KEGG) pathway analysis of differentially expressed genes identified pathways involved in cell cycle, DNA repair, gene expression, and cellular senescence as the most significantly altered pathways. **(F)** Table of several canonical SASP genes (31) that were differentially expressed in senescent A549 cells. Significance defined as –log_10_(p-adj) > 1.3. **(G)** SASP transcriptional heat map of senescent A549 with telomere dysfunction and primary AEC2s from mice with AEC2-specific telomere dysfunction (9). The log_2_-fold change is shown. Gray stripes indicate genes with no mouse homolog.

We hypothesized that expression of TRF2-DN would result in a DNA damage response and additional transcriptional changes associated with senescence. Therefore, bulk RNA sequencing was performed on control A549 cells and TRF2-DN cells 9 days after addition of doxycycline. Examination of the cluster analysis of differentially expressed genes, Pearson correlation coefficients, and principal component analysis confirms the creation of a transcriptionally distinct population of cells after the induction of TRF2-DN expression (Figure 2A and Supplemental Figures 1A,B). Consistent with previous reports focused on the effects of doxycycline (44, 45), we identified a cluster of genes that were differentially expressed due to the addition of doxycycline (not shown). We also found a significant number of genes that were differentially expressed in A549 vs. TRF2-DN in the absence of doxycycline, suggesting that low baseline expression of TRF2-DN was causing significant transcriptional changes in these cells. We focused our analysis on TRF2-DN cells to identify genes that were upregulated when these cells transitioned into senescence. Nearly 22% of all detected genes were significantly differentially expressed in this context (Figure 2B). *TERF2* (our overexpressed target) experienced the greatest increase in gene expression, followed by *SERPINE1* and *CDKN1A*—two canonical senescence genes (31) (Figure 2C). The most downregulated genes are shown in Figure 2D and include *FTL, ENO1*, and *IGFBP4* among others. Pathway analysis of our bulk RNA-seq data revealed an enrichment in pathways consistent with telomere dysfunction and disruption of the cell cycle. Notably, there was also enrichment in the cellular senescence KEGG pathway (Figure 2E). An upregulation in the canonical SASP components was also seen (Figure 2F). We validated several of the differentially expressed genes using quantitative real-time PCR with primers specific for the genes of interest and found excellent correlation with our RNA-seq data (Supplemental Figure 2). We compared our results to primary senescent murine AEC2s and found 127 (13% of upregulated murine genes) genes were upregulated in both datasets and 123 (14% of downregulated murine genes) were downregulated in both datasets (Supplemental Figure 1C). Unlike the upregulation seen in our RNA-seq dataset, very few SASP genes were upregulated in senescent murine type II alveolar epithelial cells (Figure 2G).

### Biotinylation of the Secretome

**Figure 3:**
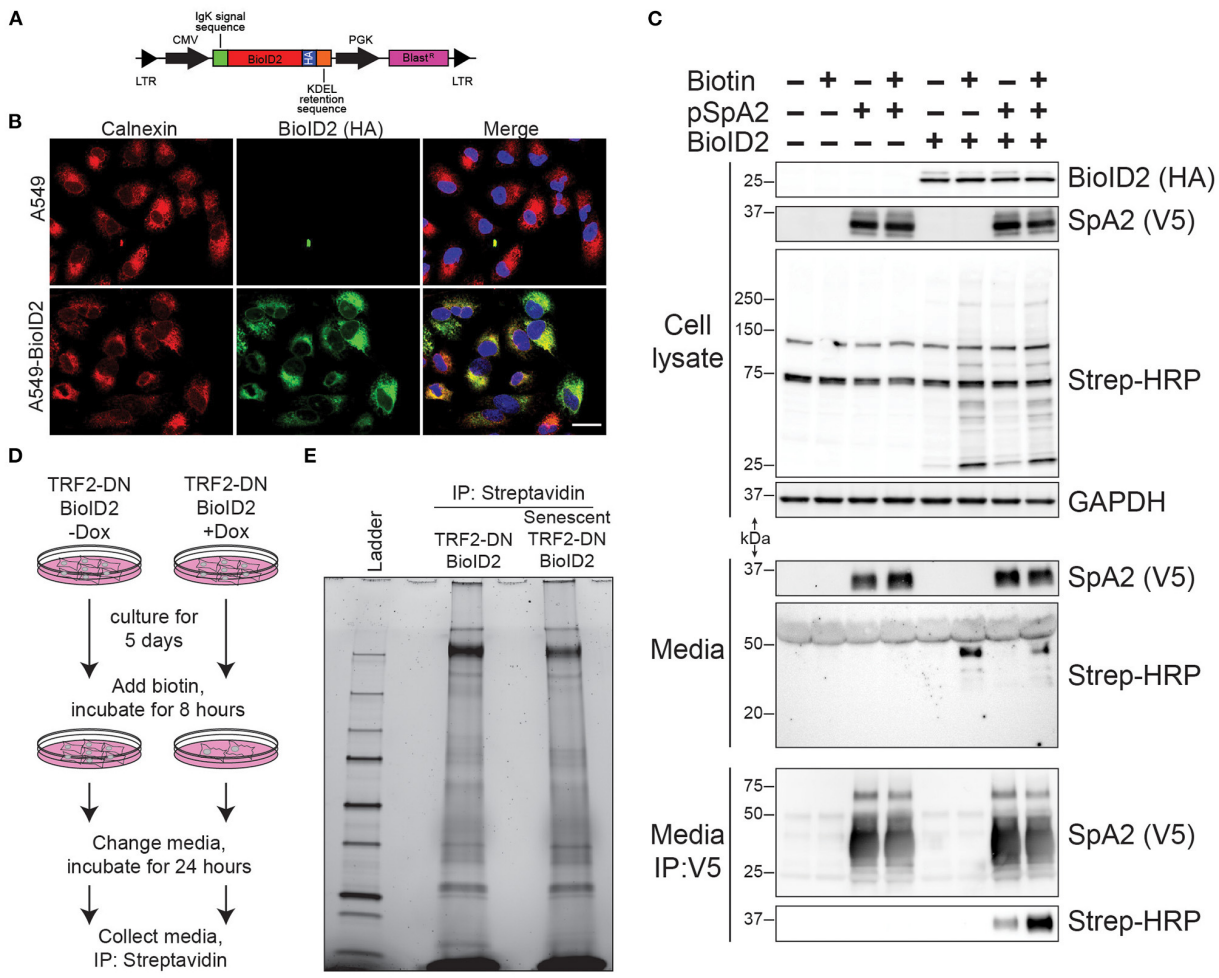
ER-targeted BioID2 to label secreted proteins. **(A)** Schematic of lentivirus expressing ER-targeted BioID2 construct. **(B)** Photomicrographs of A549 and A549-BioID2 cells showing co-localization of the BioID2 (HA-tagged; green) and the ER marker calnexin (red). Nuclei are stained with DAPI (blue). Scale bar is 25 microns. **(C)** Proof of concept demonstrating that secreted proteins are biotinylated by ER-targeted BioID2. A549 or A549 cells stable expression ER-BioID2 were transfected with a plasmid encoding a V5-SFTPA2 construct. Eighteen hours after transfection, biotin was added to some samples and the media and cell lysates were examined the next day by western blotting for SFTPA2 (V5), BioID2 (HA), or biotinylated proteins (Strep-HRP). GAPDH was a load control for cell lysates. Biotinylated proteins were detected in the media only in cells that expressed ER-BioID2 and were cultured in excess biotin. Biotinylated SFTPA2 was detected in media only when ER-BioID2 was present. **(D)** Experimental procedure for unbiased proteomic analysis of secreted proteins. Cells were cultured for 5 days in the presence of Dox to induce senescence followed by incubation with excess biotin for 8 h. After biotin labeling, cells were washed and fresh media was added. Twenty-four hours later, media was collected and biotinylated proteins were isolated by incubation with streptavidin-coated beads and analyzed by stain-free SDS-page **(E)**.

In an effort to identify senescence-associated changes in protein secretion in an unbiased manner, we next employed an endoplasmic reticulum (ER)-targeted biotin ligase (BioID2) capable of biotinylating proteins that traverse the classical secretion pathway. A lentiviral vector system was again used to stably express the ER-targeted BioID2 (Figure 3A). Confocal microscopy confirmed ER-localization of our HA-tagged BioID2 (Figure 3B). We next performed a proof-of-concept experiment to test if the ER-targeted BioID2 system was indeed functioning as anticipated. A V5-tagged surfactant protein A2 (SpA2) plasmid that has been reported to be successfully secreted by A549 cells (14) was introduced via transfection into our A549-BioID2 cell line. We first confirmed the presence of SpA2 in the transfected cell lysates. We then verified an increase in biotinylation in lysates from the BioID2 line and a further increase in biotinylation in the lysates of these cells when cultured in the presence of excess biotin. Upon blotting of the cell supernatants for the V5 epitope, SpA2 was readily detectable in the transfected lines (Figure 3C, upper panels). We probed the supernatants with streptavidin-HRP and found an increase in biotinylated proteins in the BioID2 cell lines when grown in the presence of excess biotin (Figure 3C, middle panels). Immunoprecipitation of SpA2 from supernatants followed by blotting with streptavidin-HRP demonstrated that SpA2 was being biotinylated uniquely in our A549-BioID2 system and to a greater extent when grown in the presence of excess biotin (Figure 3C, lower panels).

We next conducted an unbiased screen to identify changes in the secretome as a result of cellular senescence. We utilized our TRF2-DN-BioID2 line ± doxycycline with the addition of excess biotin to the media 5 days after the induction of senescence. After a biotin incorporation period, the media was replaced and later collected for affinity purification with streptavidin beads (Figure 3D). A portion of the streptavidin beads were eluted and evaluated using SDS-PAGE to verify protein abundance and to identify qualitative differences in protein secretion (Figure 3E). The remainder of the biotinylated protein was then analyzed via mass spectrometry in order to identify quantitative differences in secretion as a result of cellular senescence.

### Senescence-Related Changes in the Secretome

**Figure 4:**
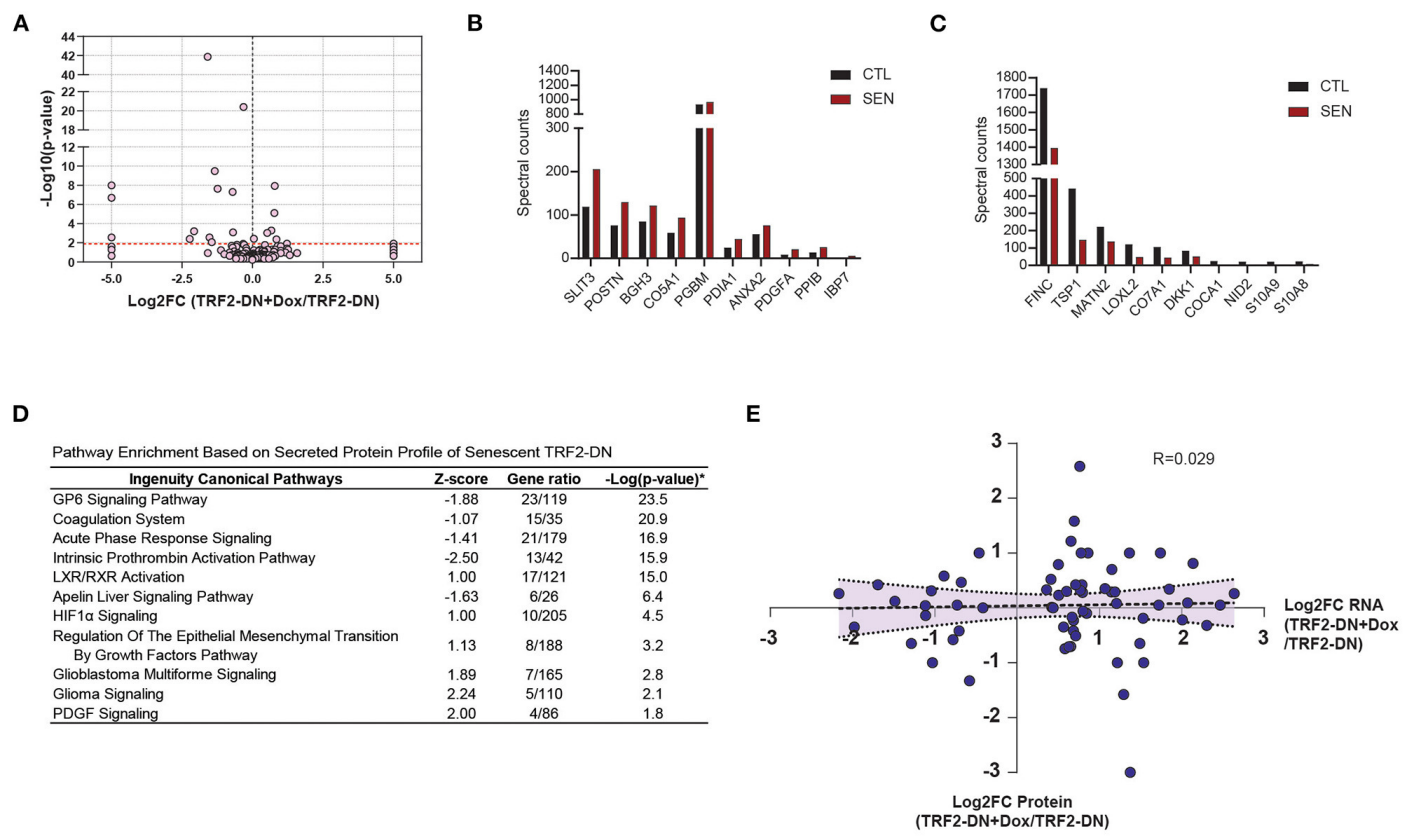
Unbiased proteomic characterization of senescent secretome. **(A)** Volcano plot showing relevant abundance of proteins in supernatant from TRF2-DN+Dox compared to TRF2-DN cells. The 10 most significantly upregulated **(B)** and downregulated **(C)** proteins between TRF2-DN (control) and TRF2-DN+Dox (senescent) cells (data are from a single experiment). **(D)** Ingenuity Canonical Pathway analysis of differentially expressed secreted proteins shows and enrichment in pathways associated with coagulation, non-specific defense, adhesion, and lipid metabolism. **(E)** Correlation of the fold-changes of RNA and protein from RNA-seq and proteomic data shows limited correlation between the two datasets.

Cumulatively, 170 unique secreted proteins were identified by LC/MS/MS for the senescent and non-senescent groups (Figure 4A). The most significantly upregulated proteins are shown in Figure 4B, which includes the SASP protein, IBP7. Fibronectin 1 (FINC) and Thrombospondin 1 (TSP1) exhibited the greatest decrease in protein expression (Figure 4C). Ingenuity Canonical Pathway analysis of the secretome revealed multiple significantly enriched pathways. Of note, these included pathways associated with coagulation, non-specific defense, adhesion, and lipid metabolism (Figure 4D). We next analyzed the relationship between transcriptional fold change and corresponding proteomic fold change in the secretome and observed a poor correlation between the two datasets (Figure 4E).

### Candidate Markers of Senescence in Idiopathic Pulmonary Fibrosis

**Figure 5:**
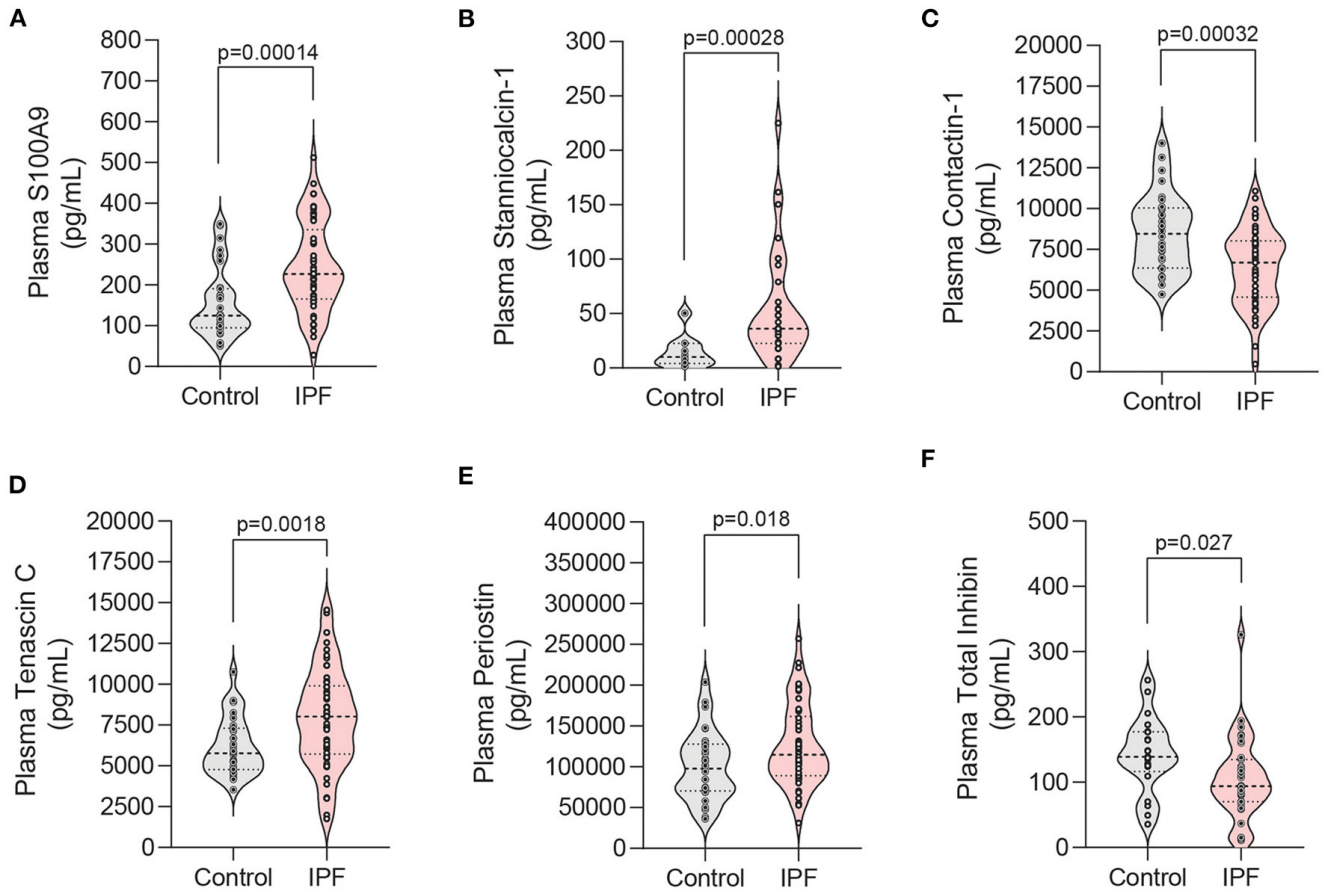
Analysis of candidate biomarkers in plasma from IPF patients and controls. **(A–F)** Violin plots of candidate biomarkers identified in RNA-seq and proteomic analyses that displayed statistically significance differences in patient plasma samples. Plasma from patients with IPF (*n* = 50) and controls (*n* = 30) was analyzed using a custom Luminex panel. Median and interquartile range is shown. Significance defined as *P* < 0.035 by Welch's *t*-test using the Benjamini-Hochberg procedure to correct for multiple testing while utilizing a pre-defined 10% false discovery rate.

Once we had established that our system closely mirrored human SASP, we next sought to evaluate its utility in identifying novel biomarkers in human plasma. Our transcriptional and proteomic data were used to rationally select 17 candidate biomarkers for evaluation in a discovery cohort of control (*n* = 30) and IPF (*n* = 50) patients. Of the 17 selected potential biomarkers, S100A9, stanniocalcin-1, contactin-1, tenascin C, periostin, and total inhibin were found to be differentially expressed between control and IPF patients (Figure 5 and Supplemental Table 1). Four of these markers (stanniocalcin-1, contactin-1, tenascin C, and total inhibin) have not been previously associated with IPF. Stanniocalcin-1 elevation displayed a trend toward an association with a lower baseline DLCO percent predicted among IPF patients, but did not reach statistical significance (*p* = 0.07) (Supplemental Table 2).

## Discussion

In an effort to better understand the pathogenesis of age and short-telomere mediated disease in the lung, we generated a human model of alveolar epithelial cell senescence. We previously investigated the transcriptional response to telomere dysfunction in primary murine AEC2s and found that few of the canonical SASP markers were expressed (9). We hypothesized that this may be due to differences in the species-specific response to telomere dysfunction and cellular senescence (36). We selected human A549 cells due to their origin in the lung epithelium and intact p53 signaling pathway. Instead of deleting TRF2, we conditionally expressed a TRF2 dominant negative (TRF2-DN) protein that has been previously reported to disrupt shelterin function (36). Consistent with disruption of telomere dysfunction and induction of a DNA-damage response, TRF2-DN expression led to accumulation of p21 and ensuing cell cycle arrest with morphologic changes consistent with the induction of cellular senescence. Despite the limitations of the A549 cell line, we reasoned that this system may provide an opportunity to explore the consequences of telomere dysfunction and cellular senescence in human alveolar epithelial cells.

We comprehensively characterized the transcriptional and secretome changes that occurred in our human telomere-induced senescence model system. Our RNA analysis demonstrated that induction of senescence in TRF2-DN leads to enrichment in pathways consistent with a telomere-based injury and to the adoption of a transcriptional SASP phenotype. When we compared our current studies to previously published findings from primary murine AEC2s, we found that in the setting of telomere-induced senescence, both human and murine cells upregulated genes related to cell cycle arrest and the DNA damage response; however, expression of SASP genes was strikingly different. Of the 60 canonical SASP genes with murine homologs, only 2 were found to be differentially expressed in the expected direction in murine cells. In contrast, 27 of the 71 canonical SASP genes were differentially expressed in the anticipated direction in human cells and several were among the top upregulated genes. Only *FAS* was similarly upregulated in both murine and human cells. These data suggest that modeling senescence and telomere dysfunction in mice may not fully recapitulate the biology of human cells and animal findings should be carefully cross-validated to ensure their translatability.

Given that previous studies have reported relatively poor correlations between human transcriptional and proteomic datasets (35, 46–48), we reasoned that an isolated transcriptional analysis of our conditionally senescent cell line would likely inadequately predict the extracellular protein and pathway changes brought on by senescence. We therefore endeavored to characterize the secretome of senescent A549 cells and adopted a recently reported system to target a biotin ligase to the endoplasmic reticulum where it would label proteins passing through the classical secretory pathway (40). We demonstrated the feasibility of the ER-targeted BioID2 system by showing that SFTPA2 is biotinylated and secreted and can be purified from cell supernatant. We then utilized our model to carry out an unbiased analysis of the secretome from senescent cells. This analysis identified 170 secreted proteins that coalesced into thematic pathways of coagulation, cholesterol homeostasis, and response to injury. A comparison of our pathway analyses following the induction of telomere dysfunction shows that transcriptional pathways primarily highlight the mechanism of injury while secretome pathways point toward downstream and paracrine effects. Consistent with prior studies, a poor correlation was found between our differential RNA and differential protein datasets (49, 50). One potential explanation is that the enrichment seen in the ubiquitin-mediated proteolysis pathway in our senescent RNA-seq dataset (data not shown) may facilitate more rapid intracellular protein degradation. Differential kinetics of protein translation and subsequent secretion in the setting of senescence is an alternative explanation. Nevertheless, the negligible interdependence between these two datasets is also not unexpected given that gene expression and protein abundance are largely uncoupled in pulmonary tissues (51). Taken together, our data suggest that transcriptional profiling alone is not sufficient to predict the secretome profile of senescent cells. Likewise, an evaluation of the secretome does not allow the inference of the intracellular signaling pathways and transcriptional aberrations initiated by telomere-mediated senescence.

Recent studies with an emphasis on aging have attempted to better characterize the many senescence-associated genes, proteins, and pathways in human disease (52, 53). Efforts have been made to highlight the varied context and cell-type specific responses to senescence (53). Similarly, we sought to utilize our telomere-mediated, alveolar epithelial cell senescence model to identify novel plasma markers of the aging associated disease, IPF. To our knowledge, this is the first report of stanniocalcin-1, contactin-1, tenascin C, and total inhibin as being differentially expressed in IPF patient plasma. Periostin (POSTN) was identified in our proteomic screen and was selected as a positive control for our study given that it had been previously reported to be upregulated in IPF patient serum (54). S100A9 was decreased in our senescent secretome data, but it had previously been shown to trend toward upregulation in IPF serum (55). We chose to evaluate this apparent discrepancy and found it to be upregulated in IPF patient plasma. Granulocytes and monocytes are reported to be the principal source of S100A9 (56), and our results suggest that senescent lung epithelial cells are not a significant source. Contactin 1 (CNTN1) was the only non-classically secreted protein evaluated in our plasma study. It was upregulated >6-fold and had the 4th most significant *p*-value for differential expression in our RNA-seq dataset, yet it was found to be downregulated in IPF plasma. Our model system correctly predicted the directionality for the remaining three differentially expressed plasma proteins lending support to its value in identifying novel classically secreted markers in IPF.

There are multiple limitations to our approach, but the data we present here highlight its value and the importance of considering potential species-specific responses to aging and telomere dysfunction. A549 is an epithelial carcinoma cell line. These cells almost certainly do not faithfully represent primary human alveolar epithelial cells and their transcriptional and secretory responses likely do not completely overlap with those of primary cells. Nevertheless, we were able to use this tractable model system to identify several candidate secreted proteins that were validated in patient plasma samples. We recognize that bronchoalveolar lavage would be a more direct measure of the epithelial secretome, but this is not clinically feasible and is an unrealistic source for potential biomarker validation. We specifically designed our experimental system to be portable to facilitate its use in studying the cell-type specific responses to telomere dysfunction in other cell lineages such as fibroblasts where telomere-based pathology has been described (57). Additionally, we expect that future studies will further delineate not only species and cell-type specific, but also context-specific responses to telomere dysfunction. Given the multiplicity of cell types in the lung and that aging and environmental factors contribute to cellular responses, additional investigations are warranted to understand how each of these cell types contribute to age-associated lung disease.

## Data Availability Statement

The datasets presented in this study can be found in online repositories. The names of the repository/repositories and accession number(s) can be found below: The RNA-seq data have been deposited to https://www.ncbi.nlm.nih.gov/geo/, GSE155941. The mass spectrometry proteomics data have been deposited to the ProteomeXchange Consortium via the PRIDE (58) partner repository with the dataset identifier PXD023381 and 10.6019/PXD023381.

## Ethics Statement

The studies involving human participants were reviewed and approved by the University of Pittsburgh Institutional Review Board and the Committee for Oversight of Research and Clinical Training Involving Decedents. The patients/participants provided their written informed consent to participate in this study.

## Author Contributions

JA and DS conceived of the project, planned the experiments, and drafted the manuscript. DS, MJ, AH, MR, and HB performed the experiments. MN and YZ analyzed the data. JL and TF provided essential reagents and protocols. JA, DS, JM, RM, and DK interpreted the findings. All authors gave feedback on the final version.

## Conflict of Interest

The authors declare that the research was conducted in the absence of any commercial or financial relationships that could be construed as a potential conflict of interest.
