# Supplementary material for: Transcriptional and Proteomic Characterization of Telomere-Induced Senescence in a Human Alveolar Epithelial Cell Line
Source: Front Med (Lausanne). 2021 Feb 9;8:600626. doi: 10.3389/fmed.2021.600626 (PMC7902064; doi:10.3389/fmed.2021.600626)
Supplement: Supplementary file 1 [file Data_Sheet_1.pdf]

Supplemental Figure 1

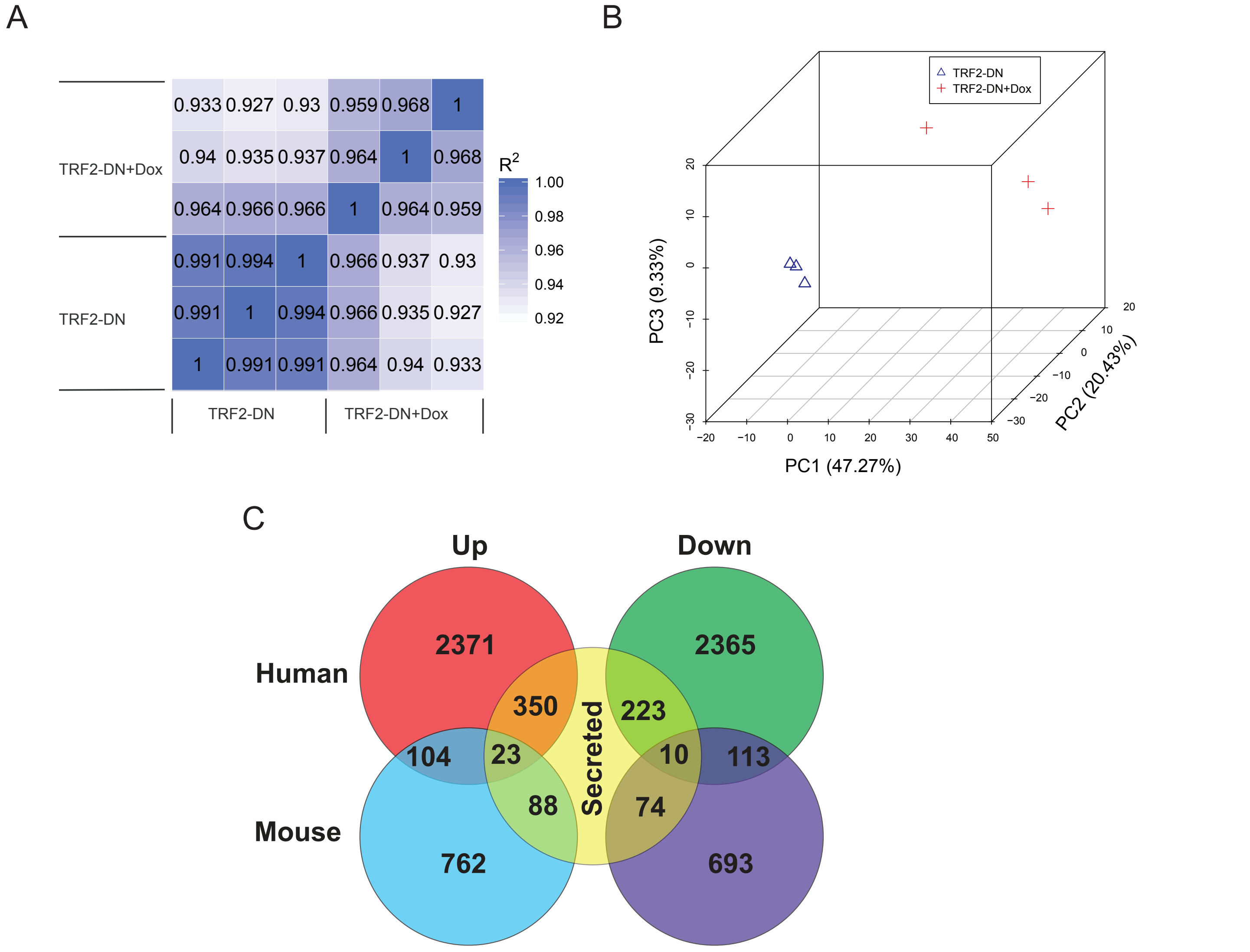

Supplemental Figure 1. Transcriptional analysis of A549-TRF2-DN cells. A) Pearson correlation coefficients amongst biologic replicates of TRF2-DN (n=3) and TRF2-DN+Dox (n=3) cells. B) Principal compenent analysis (PCA) of TRF2-DN and TRF2-DN+Dox cells show clustering amongst biologic replicates and clear differences in cells treated with doxycycline. C) Gene expression overlap between human A549 cells and primary murine type 2 alveolar epithelial cells in the setting of telomere-mediated senescence. Genes with secreted protein products from the Human Protein Atlas Secretome are highlighted in the center circle.

Supplemental Figure 2

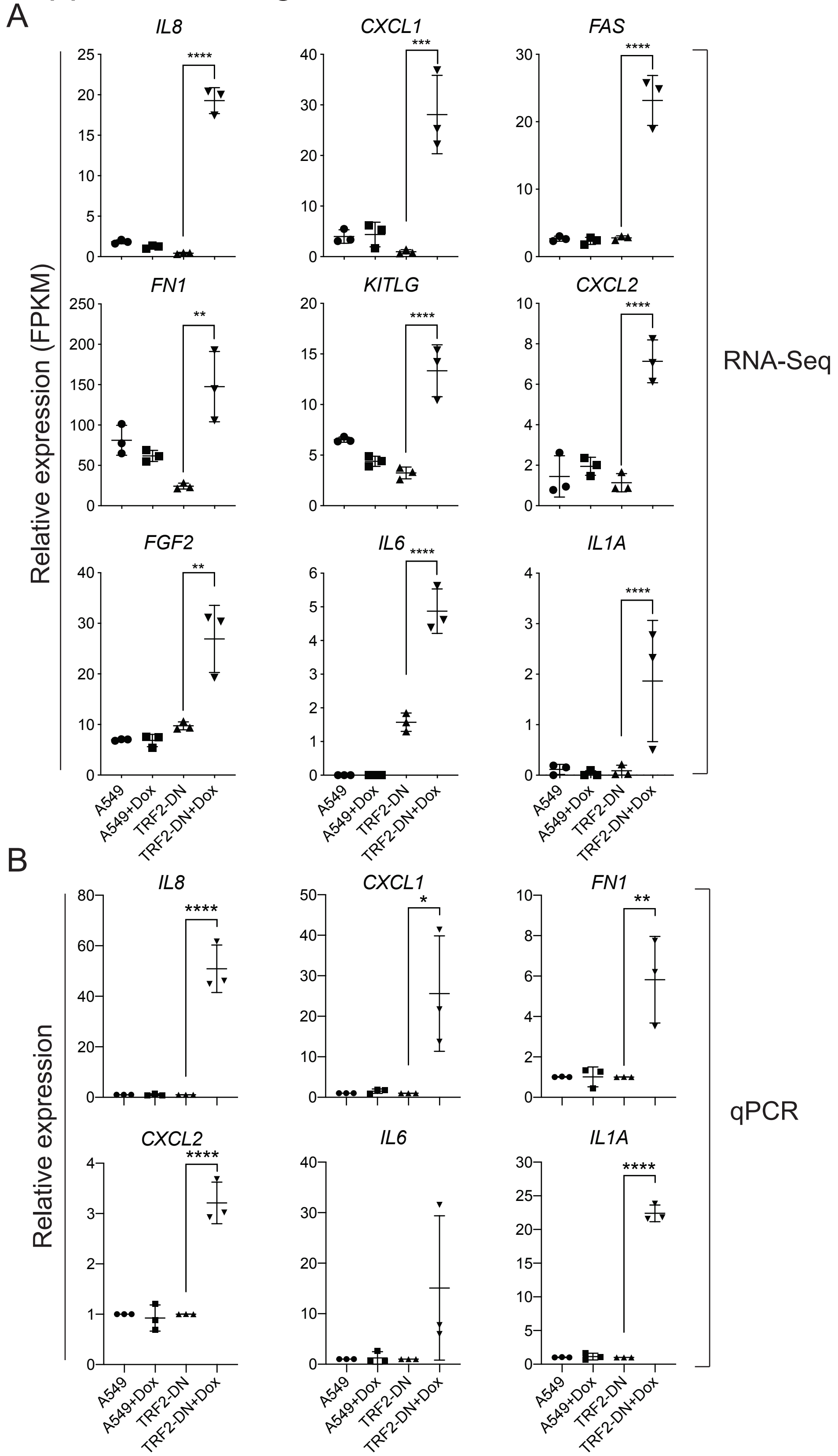

Supplemental Figure 2. Relative expression of selected senescence-associated genes. A) FPKM values were extracted from RNAseq data from each experimental treatment group 9 days after induction with doxicyline (n=3). Senescence-associated genes were selected from Coppe *Annu Rev Pathol* 2010. B) Six transcripts were selected for quantitative PCR validation. Gene expresion was normalized to *PPIA*. Groups were compared using one-way ANOVA with Tukey's test for comparing pairs of samples. Only comparisons between TRF2-DN and TRF2-DN+Dox cells is shown. *P < 0.05, **P < 0.01, ***P < 0.001, and ****P < 0.0001.

Supplemental Table 1. Comprehensive List of Proteins Evaluated in Clinical Samples

	Control (N=30)			IPF (N=50)			
Protein	Median (IQR)	OOR<	OOR>	Median (IQR)	OOR<	OOR>	P Value
S100A9	125 (97-187)	0	0	227 (166-314)	1	0	0.00014
STC1	10 (5-21)	20	0	36 (23-70)	19	0	0.00028
CNTN1	8454 (6492-9991)	0	0	6697 (4604-7962)	1	0	0.00032
Tenascin C	5776 (4828-7221)	0	0	8020 (5851-9837)	0	0	0.00175
POSTN	97932 (71581-126836)	0	0	114811 (89164-158622)	0	0	0.01794
Total Inhibin	139 (124-177)	9	0	94 (70-130)	15	0	0.02661
ANGPT1	1184 (747-2424)	0	0	703 (532-1336)	0	0	0.06307
UROK	383 (349-475)	0	0	449 (342-507)	0	0	0.07509
MK	118 (110-158)	0	0	138 (117-173)	0	0	0.08089
IBP3	447803 (410315-480029)	0	1	412923 (376295-445683)	1	1	0.08157
PDGF-DD	35 (26-48)	0	0	25 (19-45)	0	0	0.11539
PDGF-AA	70 (36-141)	0	0	41 (29-78)	0	0	0.12558
MFGM	138 (78-184)	10	0	140 (54-226)	20	0	0.18942
IBP7	11368 (8561-13501)	0	0	12370 (10078-15039)	0	0	0.29185
S100A8	5 (3-20)	24	0	25 (23-28)	48	0	0.35964
BMP7	11 (N/A)	29	0	2 (2-6)	46	0	N/A
IBP4	24 (N/A)	29	0	126 (77-189)	40	0	N/A

Supplemental Table 2. Association of Biomarkers with Baseline Pulmonary Function

Biomarker	FVC%*	DLCO%*
CNTN1	-0.03 (0.83)	-0.02 (0.88)
Total Inhibin	-0.11 (0.54)	-0.12 (0.53)
STC1	-0.20 (0.30)	-0.37 (0.07)
TNC	-0.22 (0.15)	-0.20 (0.19)
S100A9	-0.12 (0.44)	-0.24 (0.13)
POSTN	-0.07 (0.66)	0.04 (0.79)

* Correlation (P-value)
